# Determination of Cephalexin Monohydrate in Pharmaceutical Dosage Form by Stability-Indicating RP-UFLC and UV Spectroscopic Methods

**DOI:** 10.3797/scipharm.1306-07

**Published:** 2013-07-31

**Authors:** Sagar Suman Panda, Bera V. V. Ravi Kumar, Rabisankar Dash, Ganeswar Mohanta

**Affiliations:** Department of Pharmaceutical Analysis and Quality Assurance, Roland Institute of Pharmaceutical Sciences, Khodasingi, 760010, Berhampur (Odisha), India.

**Keywords:** AUC, Cefalexin, Spectroscopy, UFLC-PDA, Chromatography

## Abstract

An ultra-fast liquid chromatographic method and two UV spectroscopic methods were developed for the determination of cephalexin monohydrate in pharmaceutical dosage forms. Isocratic separation was performed on an Enable C_18_G column (250 mm × 4.6 mm i.d., 5 μm) using methanol:0.01 M TBAHS (50:50, v/v) as the mobile phase at a flow rate of 1.0 ml/min. The PDA detection wavelength was set at 254 nm. The UV spectroscopic method was performed at 261 nm and at 256–266 nm for the AUC method using a phosphate buffer (pH=5.5). The linearity was observed over a concentration range of 1.0–120 μg/ml for UFLC and both of the UV spectroscopic methods (correlation coefficient=0.999). The developed methods were validated according to ICH guidelines. The relative standard deviation values for the intraday and interday precision studies were < 2%, and the accuracy was > 99% for all of the three methods. The developed methods were used successfully for the determination of cephalexin in dry syrup formulation.

## Introduction

**Fig. 1:**
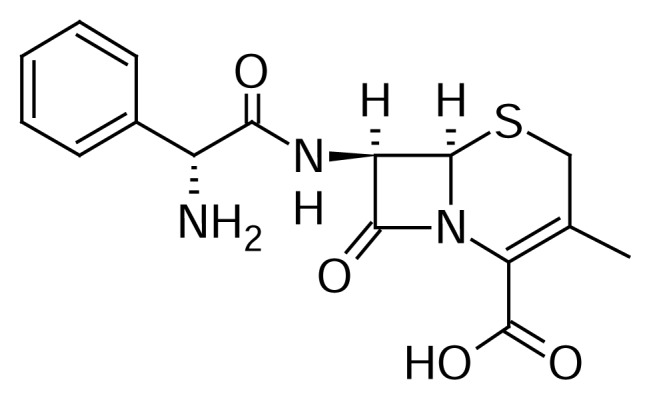
Chemical structure of cephalexin monohydrate

Cephalexin monohydrate (CEM), (7*R*)-7-(D-α-Amino-α-phenylacetamido)-3-methyl-3-cephem-4-carboxylic acid hydrate or (6*R*,7*R*)-7-{[(2*R*)-2-amino-2-phenylacetyl]amino}-3-methyl-8-oxo-5-thia-1-azabicyclo[4.2.0]oct-2-ene-2-carboxylic acid hydrate (Fig. 1) is a first generation cephalosporin antibiotic [1]. It is used in the treatment of susceptible infections of the respiratory tract, urinary tract, and skin.

CEM has been found to reduce the corrosion of mild steel in hydrochloric acid solution [2]. According to literature surveys, there are different analytical methods reported for the determination of CEM. It includes UV-Visible spectroscopy [3–12], chemiluminescence [13], near infrared spectroscopy [14], potentiometry [15], polarography [16, 17], HPLC [18–26], gel filtration chromatography [27], HPTLC [28], capillary zone electrophoresis [29], LC-MS [30, 31], and MS [32] methods. But no stability-indicating analytical methods are reported for the determination of CEM in dry syrup formulation using the mobile phase methanol:0.01M TBAHS (50:50, v/v) by UFLC (ultra-fast liquid chromatography) and UV spectrophotometric methods using a phosphate buffer of pH 5.5. So a successful attempt was made to develop and validate a fast, simple, precise, and accurate UFLC method and UV spectrophotometric methods for the determination of CEM in syrup formulation. Specificity and stability parameters for the drug were assessed according to ICH [33].

## Experimental

### Chemicals and Reagents

Cephalexin monohydrate (purity > 99.8%) was obtained as a gift sample from Cadilla Pharmaceuticals Ltd., India. Methanol (Merck Ltd., Mumbai, India) was of HPLC grade. Analytical grade sodium hydroxide, potassium dihydrogen phosphate, hydrochloric acid, and hydrogen peroxide were procured from S.D. Fine Chem. Ltd., Mumbai, India. The water for HPLC was obtained by using the TKA Water Purification System, Germany. Tetrabutylammonium hydrogen sulfate (TBAHS; Hi-Media Laboratories Ltd., Mumbai, India) was of AR grade. The dry syrup formulation containing 125 mg/5 ml of CEM was bought from the local market.

### Instrumentation

#### UFLC (Method-I)

Quantitative UFLC was performed on a binary gradient UFLC with two Shimadzu Prominence UFLC LC-20AD pumps, with a 20 μl sample injection loop (manual) and SPD M20A PDA detector. The signal was recorded and integrated using Shimadzu LC Solution Software. An Enable C18G, (250 mm × 4.6 mm i.d., particle size 5 μm) was used for separation. Chromatographic analysis was carried out at ambient temperature on the column using the methanol: 0.01 M TBAHS (50:50, v/v) as the mobile phase at a flow rate of 1.0 ml/min in isocratic mode. The 0.01 M TBAHS solution was prepared by accurately weighing 3.3954 g of TBAHS salt and dissolving it in 1000 ml of HPLC grade water. Afterwards, both the methanol and TBAHS were ultrasonicated (Enertech, India) up to 20 minutes for degassing before use. The PDA detection was carried out at 254 nm. A water bath (Thermolab, India) and UV chamber (Jain Scientific Glass Works, Ambala, India) were used for the forced degradation study of the drug.

#### UV Spectroscopy (Method-II & III)

A Shimadzu UV Visible Model 1800 double beam spectrophotometer with 10 mm matched quartz cuvettes was used for the spectral measurements. The spectrophotometer was controlled by UV Probe software which was also used to transform the UV spectra obtained. A potassium dihydrogen phosphate buffer of pH 5.5 was used as the solvent.

### Preparation of Standard and Sample Solution

Standard stock solutions of CEM were prepared by transferring 25 mg of the drug into two separate 25 ml volumetric flasks having 10 ml of diluents and were ultrasonicated for 5 minutes. Finally the volume was made up with suitable diluents, which gave 1000 μg/ml solutions. The chromatographic mobile phase and buffer solution were used as diluents for UFLC and UV spectroscopic methods, respectively.

Powder equivalent to 25 mg of CEM was accurately measured and transferred into two separate 25 ml volumetric flasks, containing 10 ml of diluents and ultrasonicated for 20 minutes; the volume was made up and mixed well. Solutions were filtered by a 0.2 μm filter to remove particulate matter, if any. The filtered solutions were properly diluted for analysis as already described. The drug present in the sample solutions was calculated by using the calibration curves. All the solutions were stored at 2–8 ºC for future use.

### Method Validation

#### Specificity

The specificity of the UFLC method was determined by checking the interference of any of the possible degradation products produced during the forced degradation study of CEM. The forced degradation of the drug was carried out with 0.1 M HCl, 0.01 M NaOH, 3% v/v H_2_O_2_, thermal (80 °C), and photolysis (365 nm) for discovering the stability nature of the drug. The degraded samples were prepared by taking suitable aliquots of the drug solution, and then undertaking the respective stress testing procedures for each solution. After the fixed time period, the stressed test solutions were diluted with the mobile phase. For every stress condition, a solution of concentration 80 μg/ml of CEM was prepared. The specific stress conditions are described as follows.

##### A: Acidic degradation condition

Acidic degradation was carried out by adding 1 ml of 0.1 M HCl, and after 45 min neutralizing the mixture by adding 0.1 M NaOH.

##### B: Alkali degradation condition

Alkali degradation was carried out by adding 1 ml of 0.01 M NaOH, and after 45 min neutralizing the mixture by adding 0.01 M HCl.

##### C: Oxidative degradation condition

Oxidative degradation was performed by exposing the drug to 1 ml of 3% (v/v) H_2_O_2_ for 45 min.

##### D: Thermal degradation condition

Thermal degradation was performed by heating the drug content at 80 °C on a thermostatically controlled water bath for 45 min.

##### E: Photolytic degradation condition

Photolytic degradation was carried out by exposing the drug content to UV light (365 nm) inside an UV chamber for 25 min.

For the UV spectroscopic method, the specificity of the method was checked for any possible interference because of the commonly used excipients in the syrup formulation.

#### Linearity

An eight-point (1.0, 5.0, 10, 20, 40, 80, 100, and 120 μg/ml) and two eleven–point (1.0, 2.0, 5.0, 10, 20, 30, 40, 50, 70, 100, and 120 μg/ml) calibration curves were prepared for the UFLC and UV spectroscopic methods, respectively. The peak area for the UFLC (Method-I) was obtained by injecting 20 μl of the drug solution into the column. For UV spectroscopic determination, the absorbance (Method-II) and AUC (area under the curve; Method-III) were measured at 261 nm and 256–266 nm, respectively. Calibration curves were plotted by taking the peak area, absorbance, and area under the curve on the y-axis and the concentration (μg /ml) on the x-axis.

#### Precision

The intraday and interday precision study was carried out to check the reproducibility of the results. A concentration of 40 μg/ml and 30 μg/ml of CEM (n=6) were analyzed to find out relative standard deviation (RSD) for UFLC and UV Spectroscopic methods, respectively.

#### Accuracy

To check the accuracy of the proposed methods, recovery studies were carried out at 80, 100, and 120% of the test concentration. The recovery study was performed three times at each level. The amount of CEM present in the sample was calculated using the calibration curves.

#### Robustness

The robustness of the UFLC method was studied by deliberately changing the method parameters like flow rate of the mobile phase, detection wavelength, and organic phase composition. A series of system suitability parameters like retention time, theoretical plates, and tailing factor were determined for each changed condition according to ICH [33]. The robustness of the UV spectroscopic method was determined by changing the slit width and carrying out a solution stability study of CEM. The sample solutions were kept at room temperature on a benchtop for 24 h and the amount of drug recovered by the developed methods was calculated.

#### Limit of Detection and Limit of Quantitation

The LOD and LOQ were determined separately according to the ICH guidelines. For the UFLC method, concentrations providing a signal-to-noise ratio 3:1 and 10:1 were considered as the LOD and LOQ, respectively. In the case of the UV-Spectroscopic method, the LOD and LOQ were determined based on 3.3 and 10 times the standard deviation of the response, respectively, divided by the slope of the calibration curves.

## Results and Discussion

### Optimization

#### UFLC (Method-I)

**Fig. 2:**
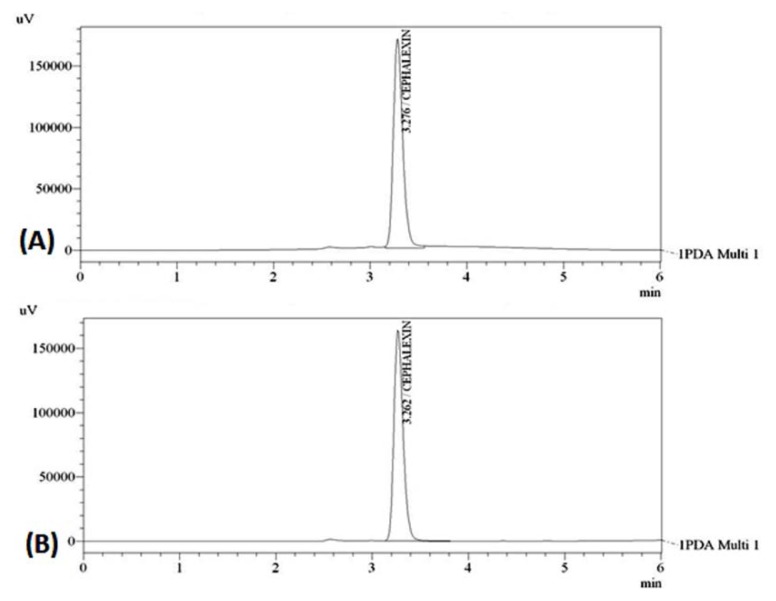
Chromatograms of CEM for Method-I (A) standard drug, (B) dry syrup formulation

Optimization of the mobile phase was carried out based on the tailing factor and theoretical plates obtained for CEM. During the trial runs, the drug was tested with different mobile phase compositions like methanol:water, methanol:0.01 M TBAHS, aceto-nitrile:water, acetonitrile:0.01 M TBAHS at various compositions (50:50, 60:40, 70:30, 75:25, v/v) and flow rates (0.8, 1.0, and 1.2 ml/min). The mobile phase consisting of methanol:0.01 M TBAHS (50:50, v/v) at a flow rate of 1.0 ml/min was selected which gave a sharp, symmetric peak for CEM. The retention time for CEM was found to be 3.276 min. The run time was 6 min. The tailing factor for CEM was found to be 1.340. PDA detection was carried out at 254 nm. The separation was carried out at room temperature. Fig. 2 (A) and (B) represents the chromatograms of the CEM standard drug and marketed dry syrup formulation, respectively.

#### UV Spectroscopy (Method-II & Method-III)

**Fig. 3:**
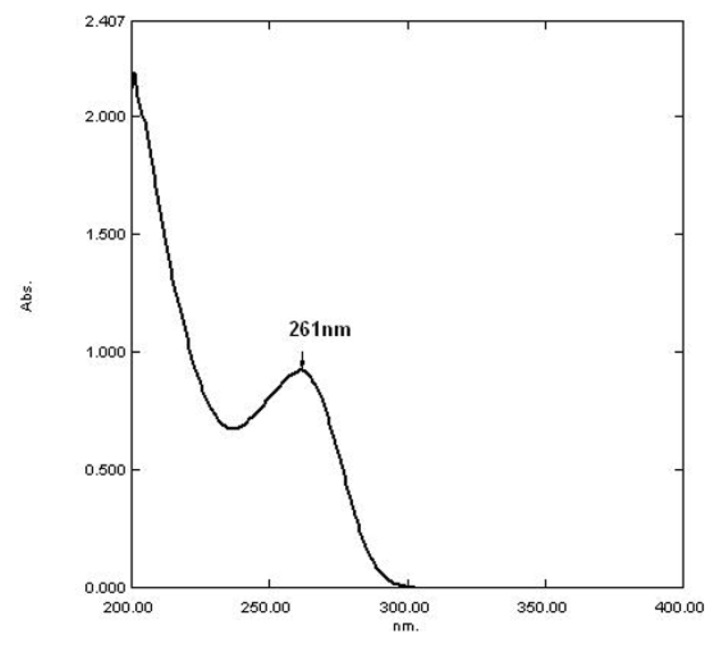
UV absorption spectrum of CEM for Method-II

**Fig. 4:**
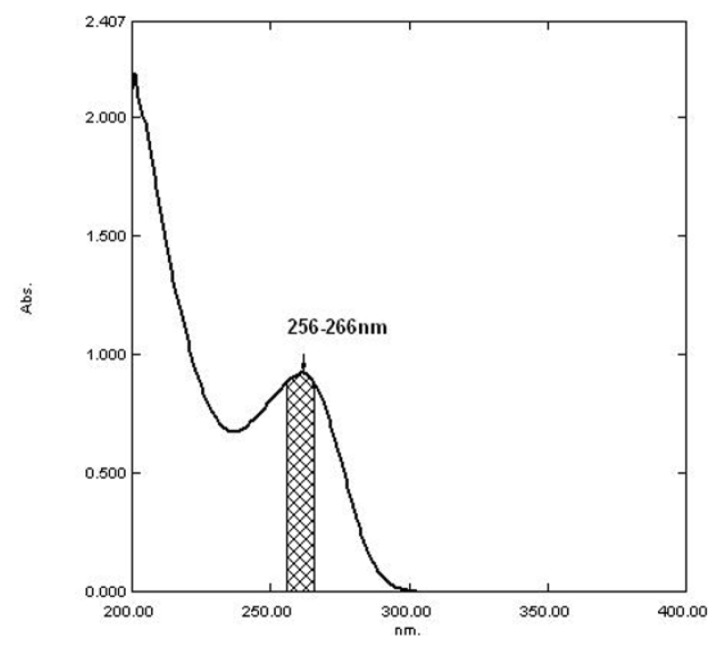
UV absorption spectrum of CEM for Method-III

The CEM in the phosphate buffer of pH 5.5 shows maximum absorbance at 261 nm (Method-II) as shown in Fig. 3. Another novel approach called the AUC method was undertaken for the calculation of the integrated value of absorbance between the two selected wavelengths λ_1_ = 256 nm and λ_2_ = 266 nm (Method-III) as shown in Fig. 4.

### Specificity

**Fig. 5:**
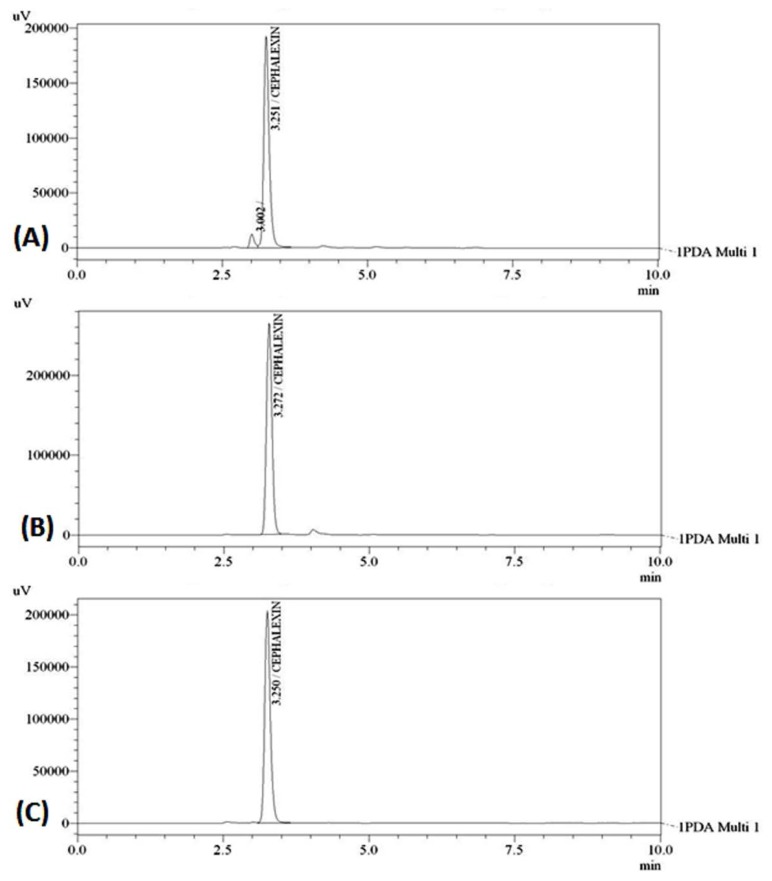
Chromatograms of CEM 80 μg/ml (A) thermal-degraded drug, (B) alkali-degraded drug, (C) photolysis-degraded drug

**Fig. 6:**
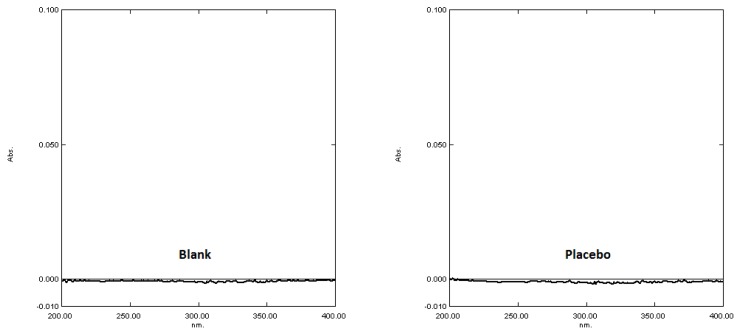
UV Spectrum of Blank Solution (left) and of Placebo Solution (right)

To evaluate the specificity, a PDA detector was applied to find out the peak purity of the chromatographic peaks obtained for the stress-treated drug solution. Peak purity results are indicative for finding out the peak homogeneity. CEM underwent severe degradation under the alkaline stress conditions by using 0.1 M NaOH and UV radiation exposure for 45 min. So the stress conditions were optimized to get moderate degradation of CEM. The modified alkaline stress was applied by using 0.01 M NaOH solution. In the case of photolysis degradation, the exposure time was decreased to 25 min. CEM showed degradation in the order of H_2_O_2_ > thermal > alkali > photolysis > acid. Fig. 5 represents typical chromatograms obtained for CEM after being subjected to thermal, alkali, and photolysis degradation conditions, respectively. The run time for each stressed drug solution was increased from 6 min to 10 min in order to find out the presence of any extra peak because of the possible degradation of CEM. But no such extra peaks were found in the chromatogram. Also, the obtained peak purity values (>0.999) suggested that there were no co-eluting or hidden peaks with the drug peak, which shows specificity and the stability-indicating nature of the method. The results for the forced degradation study are summarized in Table 1. The UV spectrums (Fig. 6) obtained for the blank and placebo show no interference due to the solvent used and presence of the commonly used excipients suggesting the specificity of the two methods.

### Linearity

The calibration curves were found to be linear over a concentration range of 1–120 μg/ml for all three methods (correlation coefficient 0.999 for all the methods). The method parameters and regression data are shown in Table 2.

### Precision

The methods were found to be precise as the RSD (%) values for the precision studies were well below 2%. The results are shown in Table 3.

### Accuracy

The accuracy of the developed methods was found out by the standard addition method. High recovery values suggest that all three methods are accurate. The results are shown in Table 3.

### Limit of Detection and Limit of Quantitation

The LOD and LOQ values shown in Table 3 suggest that the developed methods are sensitive to determine CEM.

### Robustness

The UFLC method was found to be robust under deliberate changes in the mobile phase flow rate (±0.1 mL/min), detection wavelength (±5 nm), and organic phase composition (±2%). The results of system suitability for the robustness study are shown in Table 4. For the UV spectroscopic methods, changing the slit width shows no significant effect on absorbance, indicating the robustness of the developed methods. No significant changes were obtained in the content of CEM during the solution stability studies by the developed methods. The recoveries for the solution stability by Method-I, Method-II, and Method-III were found to be 100.27%, 101.12%, and 100.65%, respectively.

### Analysis of Commercial Dry Syrup Formulation

The developed methods were successfully applied for the determination of CEM in the dry syrup formulation. The result for the assay of CEM is shown in Table 5. The assay results obtained for CEM in the syrup formulation using the UFLC and UV spectroscopic methods were compared by applying the ANOVA test, which revealed no significant differences between the values obtained by all three methods:

$${\text{F}}_{\text{calculated}}<{\text{F}}_{\text{critical}}\;(\text{P}=0.001)$$

## Conclusion

Three novel analytical methods were developed for the determination of cephalexin monohydrate (CEM). The validation study shows the methods are specific, linear, precise, accurate, and sensitive in the proposed working range. The methods were found to be fast, simple, accurate, precise, and sensitive. The excipients present in the commercial formulation were found to be non-interfering in the assay results. The methods were successfully applied for the determination of the drug in dry syrup formulation. Furthermore, the developed methods may be applied for the routine analysis of the drug in API, formulations, and dissolution medium.

## Figures and Tables

**Tab. 1:** Results of forced degradation study

Stress Applied	Degradation (%)	Peak Purity^a^
0.1M HCl	6.64	1.0000
0.01M NaOH	15.07	1.0000
3% H_2_O_2_	35.65	0.9998
80 ºC	19.39	1.0000
UV radiation at 365 nm	11.63	0.9999

a Peak purity 0.999–1.0000 indicates homogeneous peak.

**Tab. 2:** Analysis of method parameters and regression data

Parameters	Method-I^a^	Method-II^b^	Method-III^c^
Detection Wavelength, nm	254	261	256–266
Linear range, μg/ml	1–120	1–120	1–120
Slope	25893	0.022	0.224
Intercept	1764	0.003	−0.027
Correlation coefficient	0.999	0.999	0.999

a RP-UFLC Method;

b UV Spectroscopic Method;

c AUC UV Spectroscopic method.

**Tab. 3:** Summary of validation parameters

Parameters	Method-I	Method-II	Method-III
Accuracy(recovery),%	100.17–101.22	99.23–100.93	99.96–101.7
Precision(RSD),%			
Intraday	0.64	0.14	0.22
Interday	0.98	0.25	0.25
LOD, μg/ml	0.24	0.28	–
LOQ, μg/ml	0.78	0.86	–

**Tab. 4:** Robustness results

Parameter	Retention Time (min)	Theoretical Plates	Tailing Factor
Flow rate (ml/min)			
0.9	3.615	4736	1.336
1.0	3.272	4338	1.340
1.1	2.974	4277	1.332
Wavelength (nm)			
249	3.272	4344	1.340
254	3.272	4338	1.340
259	3.272	4339	1.340
Methanol (%)			
48	3.400	4801	1.241
50	3.272	4338	1.340
52	3.173	4995	1.352

**Tab. 5:** Assay of syrup formulation

Formulation Label Claim	Recovery^a^ (%)± SD, RSD (%)		
	
	Method-I	Method-II	Method-III
Each 5ml contains	100.38 ± 0.14,	100.14 ± 0.16,	100.85 ± 0.017,
≈ 125mg of drug	0.14	0.16	0.017

a average of three determinations at each level.
